# Supplementary material for: Factors associated with premarital HIV testing among married women in Ethiopia
Source: PLoS One. 2020 Aug 3;15(8):e0235830. doi: 10.1371/journal.pone.0235830 (PMC7398550; doi:10.1371/journal.pone.0235830)
Supplement: S1 Checklist — (DOC) [file pone.0235830.s001.doc]

STROBE Statement—Checklist of items that should be included in reports of ***cross-sectional studies***

|  | Item No | Recommendation |
| --- | --- | --- |
| **Title and abstract** | 1 | ***Factors associated with premarital HIV testing among married women in Ethiopia.***  Mohammed Ahmed, Abdu Seid |
|  |  | **Background:** Premarital HIV testing is the key entry point to prevention, care, treatment, and support services, where people learn their HIV status and its implications to make informed decisions about their health. This study was, therefore, conducted to identify factors associated with premarital HIV testing among married women in Ethiopia.  **Methods:** A cross-sectional study design was used, and secondary data analysis was done using 2016 Ethiopian demographic health survey (EDHS). Two-stage stratified cluster sampling technique was used. The data were analyzed by SPSS version 20. Frequencies and weighted percentage of the variables, and second-order Rao-Scott statistic were computed. Multivariate logistic regression analysis was performed to control confounders and to identify predictors of premarital HIV testing. Adjusted odds ratio was considered to declare statistically significant association.  **Result:** The total sample comprised 9602 married women. In this study, the odds of premarital HIV testing were associated with being urban residents, attended primary education, Secondary education**,** higher education**,** had media access, being rich, and richest**,** had know the place of HIV testing**,** had discriminatory attitude to PLHIV, being khat chewer, and alcohol drinker.  **Conclusion:**  The study concluded that being urban resident, attending education (primary, secondary, higher), media access, improved wealth index, knowing place for HIV testing, chewing khat, drinking alcohol, and having discriminatory attitude towards PLHIV were positively associated with premarital HIV testing.  **Keywords**: Premarital HIV testing, Married, Women, Ethiopia Demographic and Health survey. |
| Introduction | | |
| Background/rationale | 2 | Step up prevention activities require a thorough understanding of the HIV epidemic character, modes of transmission and populations affected as these inform the extent to which evidence based modalities can be adapted and pooled to substantially reduce HIV transmission, which is critical in continuing the path to avert epidemic trajectory .  In a nutshell, premarital testing is the best solution for prevention, care, treatment, and support services . Make out and intervening thus factors enable for undertakes premarital HIV testing, which leads to trim down HIV acquisition among couples. Therefore, the endeavor of this study was to identify factors associated with premarital HIV testing among married women in Ethiopia. |
| Objectives | 3 | The aim of this study was to identify factors associated with knowledge about mother to child transmission of HIV among married men using 2016 Ethiopian demographic health survey. |
| Methods | | |
| Study design | 4 | A community-based cross-sectional study design was used, and a nationally representative secondary data analysis was done on the 2016 EDHS. The samples were selected using a two-stage stratified cluster sampling technique. |
| Setting | 5 | Ethiopia |
| Participants | 6 | Married women aged 15-49 years |
| Variables | 7 | The main outcome of interest was self-reported history of premarital HIV testing among married women (yes/no).The independent variables were selected based on literature review which deemed to be the factors associated with premarital HIV testing and includes age, education, type of residence, occupation, wealth index, , media access , Knowing place for HIV testing , comprehensive knowledge on HIV, discriminatory attitude to HIV, khat chewing, and alcohol drinking. |
| Data sources/ measurement | 8* | The source of data for this study is EDHS 2016 which was collected from January 18, 2016, to June 27, 2016. |
| Bias | 9 | N/A |
| Study size | 10 | 9602 |
| Quantitative variables | 11 | The data were analyzed by SPSS version 20. Frequencies and weighted percentage of the variables were calculated |
| Statistical methods | 12 | The data were analyzed using Statistical Package for Social Science (SPSS) version 20.Multivariate logistic regression analysis was performed to control confounders and to identify predictors about premarital HIV testing. All independent variables were entered in the multivariate logistic regression model irrespective of the p-values in the statistical significance in the bivariate analysis. Adjusted odds ratios (AOR) were used to declare statistically significant association |
|  |  | (*b*) N/A |
|  |  | (*c*) N/A |
|  |  | (*d*) N/A |
|  |  | (*e*) N/A |
| Results | | |
| Participants | 13* | (a) A total of 9602 sub-sample of married women within the EDHS 2016 were included and analyzed |
|  |  | (b) N/A |
|  |  | (c) N/A |
| Descriptive data | 14* | (a) Majority of respondents (84.2 %) were rural resident and 23.5 % were in the age between 25-29 years. Out of these subsample, 61.8 % of the respondent didn’t attend education. Only 96.8 % of married women’s didn’t have comprehensive knowledge for HIV, and 62.2% didn’t have access to media. |
|  |  | (b) N/A |
| Outcome data | 15* | Premarital HIV testing |
| Main results | 16 | (*a*) Being rural resident, attending education (primary, secondary, higher), media access, being rich and richest, knowing place for HIV testing, chewing khat, alcohol drinking, and having discriminatory attitude towards HIV were positively associated with premarital HIV testing.. |
|  |  | (*b*) N/A |
|  |  | (*c*) N/A |
| Other analyses | 17 | N/A |
| Discussion | | |
| Key results | 18 | In this study, premarital HIV testing was positively associated with residence, educational status, media access, wealth index, knowing the place for HIV testing, chewing khat, drinking alcohol, and having discriminatory attitude towards PLHIV. |
| Limitations | 19 | We cannot assign causations to any of the associations between the identified factors and the outcomes of interest due to cross sectional data |
| Interpretation | 20 | The study concluded that being urban resident, attending education (primary, secondary, higher), media access, improved wealth index, knowing place for HIV testing, chewing khat, drinking alcohol and having discriminatory attitude towards PLHIV were positively associated with premarital HIV testing. The Ethiopian government needs to step up efforts to expand education for all Women. Advancing access to HIV testing for rural women may also increase premarital HIV testing services uptake. Further qualitative research need to be done to assess the relationship between discriminatory attitude towards PLHIV and premarital HIV testing. |
| Generalisability | 21 | Generalize to Ethiopian Women population |
| Other information | | |
| Funding | 22 | N/A |
