# Supplementary material for: Factors associated with premarital HIV testing among married women in Ethiopia
Source: PLoS One. 2020 Aug 3;15(8):e0235830. doi: 10.1371/journal.pone.0235830 (PMC7398550; doi:10.1371/journal.pone.0235830)
Supplement: Attachment — Submitted filename: Response to reviewers.docx [file pone.0235830.s003.docx]

Thank you very much for PLOS one editorial office, academic editors, as well as reviewers of this manuscript entitled with **factors associated with premarital HIV testing among married women in Ethiopia** for their astonished effort.

The written documents below explained point by point response for respective editors and reviewers

**Reviewer 1 comments for authors:**

As per PLOS instructions the authors need to “describe where the data maybe found in full sentences” they provide this information in their response to the reviewer’s comments, but it is not provided in the article submission”. The manuscript continues to require to editing for English.

**Author response:** For this analysis, we used the USAID–DHS program 2016 Ethiopian demographic and health survey data set. To request the same or different data for another purpose, a new research project request should be submitted to the DHS program here: <https://dhsprogram.com/data/Access-Instructions.cfm>. The DHS Program will normally review all data requests within 24 – 48 hours (during working days) and provide notification if access has been granted, or additional project information is needed before access can be granted. After receiving permission, the researcher can login and select the specific data in the format they prefer. The above information is included and attached in the revised manuscript.

The English language, grammar and spelling errors were corrected by linguist.
